# Extracorporeal Membrane Oxygenation (ECMO)—A Lifesaving Technology. Review and Single-center Experience

**DOI:** 10.5041/RMMJ.10363

**Published:** 2019-04-18

**Authors:** Maged Makhoul, Keren Bitton-Worms, Zvi Adler, Ayman Saeed, Oved Cohen, Gil Bolotin

**Affiliations:** 1 Department of Cardiac Surgery, Rambam Health Care Campus, Haifa, Israel; 2 The Ruth & Bruce Rappaport Faculty of Medicine, Technion–Israel Institute of Technology, Haifa, Israel

**Keywords:** Extracorporeal cardiopulmonary resuscitation, extracorporeal membrane oxygenation, veno-arterial ECMO, veno-venous ECMO

## Abstract

**Objective:**

Extracorporeal membrane oxygenation is used to bypass the cardiopulmonary system in a severe heart or/and lung failure, mainly in intractable conditions where all other therapy options fail or are unfeasible. Extracorporeal membrane oxygenation (ECMO) is a well-established therapeutic option in such circumstances for neonatal, pediatric, and adult patients. Managing a patient with ECMO requires dedicated and specific management. The importance and necessity of this essential technology in life-threatening cardio-respiratory rescue prompted Rambam Health Care Campus to implement it and make it available as a service to the population in northern Israel. This article includes a brief review of extracorporeal life support and a report of our single-center experience since the establishment of the service.

**Methods:**

The ECMO unit was established in 2014 under the responsibility of the Cardiac Surgery Department. The ECMO service was initiated by a well-planned program with consideration of all aspects including economics, education and training, the specialist team and equipment needed, strategies for medication, and ethical challenges.

**Results:**

Between February 2014 and May 2018, 65 patients were treated with ECMO; 43 patients received veno-arterial ECMO for cardiac support (66%), while 22 received veno-venous ECMO for respiratory support (34%). The in-hospital mortality was 56%.

**Conclusions:**

Extracorporeal membrane oxygenation is an effective therapy that is constantly growing in use and provides a therapy that can replace previous options. To establish such a service requires a planned program and concerted effort. Our single-center experience presented a good learning curve and showed the feasibility as well as the efficacy of the ECMO procedure in life-threatening conditions.

## INTRODUCTION

In the era of percutaneous extracorporeal cardiopulmonary support devices, extracorporeal membrane oxygenation (ECMO) has become an important tool in the modern intensive care unit arsenal. It provides cardiac and respiratory support in cases of acute deterioration regardless of the etiology.

## HISTORY AND DEVELOPMENT

In the mid-1950s, Gibbon discovered the benefits of the disk-oxygenator, providing an important tool for prolonged cardiac operations. Subsequently, extracorporeal oxygenation was developed to support patients with severe cardiopulmonary dysfunction. In 1961, Callaghan et al. documented the first animal ECMO experiment on preterm neonate dogs with acute respiratory distress syndrome (ARDS).^1^ In 1979, 90% mortality was reported in a randomized control study of adult patients with ARDS.^2^ With the advanced technological development of the silicon oxygenator and dialyzer, improved monitoring and control of gas and blood flow, and a better understanding of ECMO physiology, survival rates have dramatically improved, as shown in several studies, mainly during the H1N1 influenza pandemic.^3^–^6^ According to the Extracorporeal Life Support Organization (ELSO), almost 100,000 ECMO runs were reported worldwide at more than 300 centers, with 68% ECMO survivors and 56% surviving to discharge.^7^

## ECMO SYSTEM AND CANNULATIONS

Extracorporeal membrane oxygenation is used to bypass the cardiopulmonary system, mainly in intractable conditions where all other therapy options fail or are unfeasible. The idea of bypassing the heart and lungs is to supply oxygenated blood directly to the body tissues independently, giving the heart and lungs enough time to recover.

The decision whether to connect a patient to veno-arterial (VA) or veno-venous (VV) ECMO depends on the patient’s illness and situation. This system provides short-term mechanical circulatory support in varied acute life-threatening conditions, and it provides proper and effective body tissue oxygenation in cases of respiratory failure such as ARDS. In these cases, VV ECMO bypasses the failing lungs. It also provides circulatory and hemodynamic support in cases of cardiac failure like in post-myocardial infarction and cardiogenic shock. In these cases, VA ECMO is used to bypass the heart–lung system.

Veno-arterial ECMO cannulation is achieved by introducing a large venous cannula to the venous system and a smaller arterial cannula to the arterial system. The blood exits the body driven by gravity and the low negative pressure generated by the centrifugal pump, passes through the silicon heparin-coated tubes to the centrifugal pump, then through the polymethylpentene hollow fiber membrane oxygenator where gas exchange takes place, before returning the now oxygenated blood to the arterial system.

In case of VV ECMO, two large venous cannulas are introduced to the venous system; one pulls the blood from the vena cava system to the ECMO circulation, while the other drives the oxygenated blood back to the right atrium.

Several other modes of ECMO system cannulation are described involving different arterio-venous combinations and cannulation sites, depending on the situation. The most commonly used combinations are the veno-venous-arterial (VVA) and the veno-arterial-venous (VAV) cannulations. The VVA initially operates as a VV, with an arterial line added as needed, while the VAV is initially a VA with the venous line added as needed. These combinations allow physicians to better control the blood outflow distribution, pressures, and, ultimately, the hemodynamics. The so-called “hybrid ECMO modes” are used mainly for dynamic ECMO patients where the site of cannulations or the blood flow and distributions need to be modified in accordance with the patient’s situation.

## INDICATIONS AND CONTRAINDICATIONS

Although ECMO is a life-saving procedure, it is dangerous and risky. Therefore, careful and proper patient selection as well as following appropriate and well-established implant procedure techniques is of vital importance. Patients eligible for ECMO are generally critically ill with progressing cardiopulmonary condition; timing is crucial, and a decision whether to connect these patients to ECMO must be made quickly and accurately. During the patient examination, ECMO physicians usually rapidly examine the patients, considering some indications and ruling out some contraindications before making a decision.

Hypoxic respiratory failure and ARDS are the most common indications for VV ECMO. According to ELSO,^7^ ECMO should be considered if the mortality risk is 50% and is indicated if the mortality risk is 80% despite optimal mechanical ventilation. Treating these conditions with ECMO is done only after all other therapeutic options for the failing lungs have been exhausted, such as ideal mechanical ventilation, nitric oxide, prone positioning, and other lung recruitment maneuvers. Other indications are CO_2_ retention on optimal mechanical ventilation, severe air leak syndrome, need for intubation in a patient on the lung transplant list, and immediate respiratory collapses like in pulmonary embolism or blocked airway.

As for VA ECMO, the indications are the conditions that lead to cardiac insult and consequently cardiogenic shock and heart failure, such as myocardial infarction, myocarditis, cardiomyopathies, and others. Other indications for VA ECMO are failure to wean from cardiopulmonary bypass machine in open-heart surgery, refractory cardiopulmonary resuscitation (CPR), or as a bridge to assist device or heart transplant.

According to ELSO, there are no absolute contraindications for ECMO use; the physician calculates the risks and benefits for each patient individually and decides whether or not to initiate ECMO. However, some conditions may predict worse outcome due to poor prognosis despite ECMO and are considered relative contraindications, for example, prolonged (>7 days) high ventilation settings, prior conditions with poor prognosis (e.g. malignancies, major bleedings, and in the elderly, although there is no specific age contraindication) and more (see euroelso.net for details).

## EXTRACORPOREAL CARDIOPULMONARY RESUSCITATION

Cardiopulmonary resuscitation (CPR) is a challenging and life-threatening situation. According to Cardiac Arrest Registry to Enhance Survival (CARES), only 10% of patients survive out-of-hospital cardiac arrest.^8^

After prolonged and refractory CPR, use of ECMO in an emergency setup can provide adequate blood/ tissue oxygenation regardless of cardiac function and rhythm.

The benefit of extracorporeal cardiopulmonary resuscitation (ECPR) has been described in previously published articles in terms of improving survival and neurological outcome.^9^–^13^ Shockable initial rhythm and lower low-flow time were associated with better outcomes.^12^,^14^

Unlike other ECMO indications, ECPR demands faster decisions; while the patient’s brain and heart are suffering during CPR, the physician needs to assess the situation and indicate treatment strategy, and decide whether to initiate ECMO or continue conventional CPR. As previously mentioned, advanced age is not an absolute contraindication for ECMO, although an association between elderly and higher mortality rates has been shown.^15^ For example, further consideration is warranted when considering using ECMO in an older patient who has favorable predictors for a positive outcome. On the other hand, young patients with unfavorable predictors are often treated with ECMO due to their younger age. Could it be a human empathy or a professional dilemma?

## EXPERIENCE IN THE RAMBAM HEALTH CARE CAMPUS

Until February 2014 the ECMO service was not available in northern Israel. Rambam Health Care Campus is the largest medical center in northern Israel serving the over 2 million residents. We report here our single-center experience with an ECMO system implementation.

The ECMO unit was established in February 2014 under the responsibility of the Cardiac Surgery Department. The system is used to treat both adult and pediatric patients. The program was initiated by intensive formal (didactic course) and practical training in other hospitals with strong experience of ECMO service, carried out by surgeons, nurses, perfusionists, and technicians. The team chosen to serve on the ECMO team had a strong critical care background in neonatal, pediatric, or adult critical care. The director of the new unit was a cardiac surgeon, and he took professional responsibility for every aspect of the service.

We started with one available MAQUET® system (ROTAFLOW Centrifugal Pump head, Quadrox D membrane, Getinge Group, Rastatt, Germany), a second system was purchased soon after, and a third system was recently installed.

**Figure 1:**
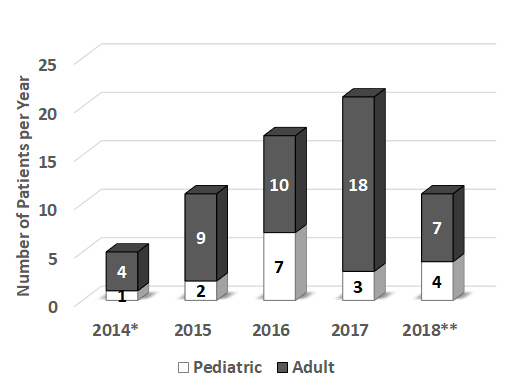
Number of Adult and Pediatric Patients Treated with Extracorporeal Membrane Oxygenation (ECMO) Annually at Rambam. * Data from February–December, 2014; **Data from January–May, 2018.

From February 2014 to May 2018, ECMO was required for 65 patients in our center. Most of the patients were adults (48/65) ranging from 18 to 78 years, who were treated in the cardiac surgery intensive care unit (ICU); the rest were neonatal and pediatric patients (17/65) ranging from 6 days to 14 years, who were treated in the pediatric ICU (Figure 1). The number of patients treated with ECMO has increased annually due to an improvement in the professional capabilities and the capacity of the ECMO unit (Figure 1).

**Figure 2:**
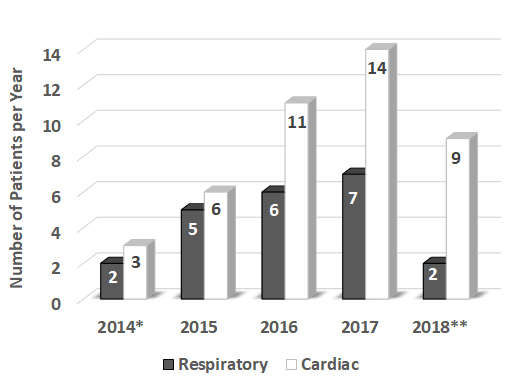
Number of Patients Treated with Extracorporeal Membrane Oxygenation (ECMO) for Respiratory or Cardiac Support per Year at Rambam. * Data from February–December, 2014; **Data from January–May, 2018.

**Figure 3:**
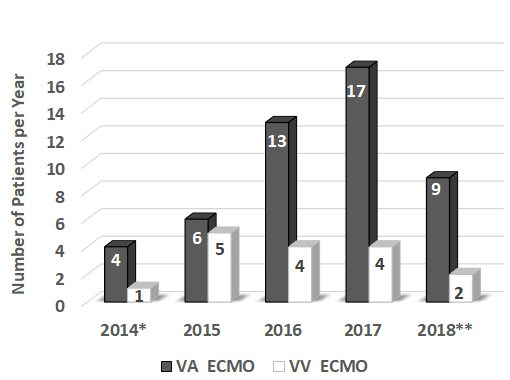
Number of Patients Treated with Veno-venous (VV) or Veno-arterial (VA) Extracorporeal Membrane Oxygenation (ECMO) Annually at Rambam. * Data from February–December, 2014; **Data from January–May, 2018.

**Figure 4:**
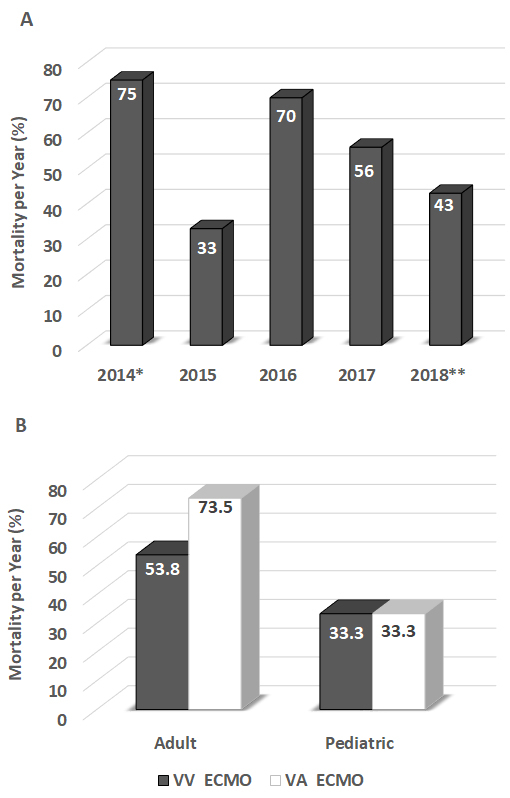
Mortality Percentages with Extracorporeal Membrane Oxygenation (ECMO) Treatment at Rambam. **A:** Percentages per year, 2014–2018. **B:** Percentages for adult and pediatric patients, for VV ECMO and VA ECMO. VV, veno-venous-arterial; VA, veno-arterial. * Data from February–December, 2014; **Data from January–May, 2018.

Indications for ECMO were divided into cardiac and respiratory support; 66% of our patients received ECMO for cardiac support, while 34% received ECMO for respiratory support (Figure 2). A wide range of clinical situations prompted the initiation of ECMO in our patient population: massive pulmonary embolism, ischemic cardiomyopathy, viral pneumonia, poisoning, snake bite, trauma, and more. The patients were treated with VV ECMO (24.6%, 16/65) or VA ECMO (75.4%, 49/65) (Figure 3). The average duration of ECMO connection was 7.8 days (±9.6), and duration of hospital stay was 21.4 days (±23.7). Among the adult patients only, the mortality was 56% during 2017 and 43% during the first 5 months of 2018 (Figure 4A). The mortality of adult patients receiving VA ECMO was higher (73.5%) compared to that of adults receiving VV ECMO (53.8%). The pediatric patients showed the same level (33%) of mortality with VV ECMO and VA ECMO mode (Figure 4B).

Immediately after establishing the service, we treated patients outside of the hospital and were able to perform cannulation outside the hospital and then transport the patients to our center on ECMO.

Twenty-six patients (40%) were referred from other hospitals in Northern Israel. For example:

- A 19-year-old student transferred from the Palestinian authority for VV ECMO after severe lung contusion.
- A 40-year-old female in anaphylactic and cardiogenic shock after a bee sting.
- An 18-year-old Druse student from the Golan heights referred for VV ECMO after organic sulfur intoxication.

The three patients above, and all the others treated, represent dramatic cases in extreme life-threatening conditions, who were rescued using the ECMO system.

## CONCLUSION

Our single-center experience presented a good learning curve together with the subsequent good results of treated patients who were weaned from the ECMO and continue to show improving results.

Today, our center exemplifies a well-established ECMO service staffed by a specialist ECMO team, with the needed programs, protocols, and standard of care in place, and the ability to treat patients off-site—all available 24 hours/day. Altogether, within a very short time, Rambam Health Care Campus has developed a service providing an invaluable and essential tool to save lives.

## Abbreviations

ARDS: acute respiratory distress syndrome
CPR: cardiopulmonary resuscitation
ECMO: extracorporeal membrane oxygenation
ECPR: extracorporeal cardiopulmonary resuscitation
ELSO: Extracorporeal Life Support Organization
VA: veno-arterial
VAV: veno-arterial-venous
VV: veno-venous
VVA: veno-venous-arterial.
